# Long-term Consequences of COVID-19 and the Pandemic: Protocol for a Web-Based, Longitudinal Observational Study (DEFEAT)

**DOI:** 10.2196/38718

**Published:** 2022-10-26

**Authors:** Marie Mikuteit, Stephanie Heinemann, Sascha Roder, Jacqueline Niewolik, Dominik Schröder, Kai Vahldiek, Sandra Klawitter, Anne Cossmann, Torsten Bergemann, Chantal Degen, Frank Klawonn, Georg Martin Norbert Behrens, Frank Müller, Alexandra Dopfer-Jablonka, Sandra Steffens

**Affiliations:** 1 Department for Rheumatology and Immunology Hannover Medical School Hannover Germany; 2 Deans' Office, Curricular Development Hannover Medical School Hannover Germany; 3 Department of General Practice University Medical Center Göttingen Göttingen Germany; 4 Department of Computer Science Ostfalia University of Applied Sciences Wolfenbüttel Germany; 5 Department of Otolaryngology Hannover Medical School Hannover Germany; 6 Biostatistics Research Group Helholtz Center for Infection Research Braunschweig Germany; 7 German Center for Infection Research Partner site Hannover-Braunschweig Germany

**Keywords:** SARS-CoV-2, COVID-19, long COVID, post–COVID-19, long haulers, pandemic, long-term effects, symptoms, long-term, risk factors, Germany, population study, quality of life, social participation, engagement

## Abstract

**Background:**

With population-wide vaccination availability, the global COVID-19 pandemic entered a new phase. Despite vaccination status, some people who were infected with SARS-CoV-2 experience long-term symptoms.

**Objective:**

In this study, we aim to characterize the long-term effects of SARS-CoV-2 infection and the pandemic. We also aim to build symptom clusters and determine risk factors for developing long COVID symptoms. Furthermore, we assess social participation and health-related quality of life in patients with long COVID and in the general population during a global pandemic.

**Methods:**

With a mixed-methods, web-based approach, we aim to recruit 2000 people in Germany who are older than 18 years and can provide informed consent. In the quantitative arm of the study, we identify symptoms of and predictive factors for long COVID manifestations with cluster analysis and assess social participation during the pandemic with standardized questionnaires. The qualitative arm of the study uses individual interviews and focus group discussions to better understand the illness experience of persons who experience long COVID.

**Results:**

Recruitment started in September 2021. Up until July 2022, we recruited approximately 4500 participants via our web-based database.

**Conclusions:**

This study aims to build an innovative, patient-centered, web-based research platform appropriate for the pandemic by minimizing physical contact between study personnel and participants. All study activities are designed to better understand the long COVID syndrome, social participation during the pandemic, and the illness experiences of persons affected by long COVID.

**Trial Registration:**

German Clinical Trial Registry DRKS00026007; https://tinyurl.com/yh282fkt

**International Registered Report Identifier (IRRID):**

DERR1-10.2196/38718

## Introduction

### Overview

In the beginning of the SARS-CoV-2 pandemic, lockdown and restrictions were the main measures to control further transmission of the virus. In 2021, we entered a new era of the pandemic, with several effective vaccines. Politics, society, and science developed and established effective measures for the control of the transmission of the virus. Nevertheless, more than 320 million people were infected worldwide until now, and more than 5 million people died because of the infection [1]. Approximately 10% of the infected people report symptoms 4 to 12 weeks after the COVID-19 infection, now called “Long COVID” or “Post COVID“ syndrome (hereafter: long COVID [2]). The symptoms after a SARS-CoV-2 infection are various; a study from the United Kingdom reported 55 different long-term effects [3]. In the German guideline, different symptoms of long COVID are described, such as headache, shortness of breath, cough, dysosmia or anosmia, cognitive impairment or brain fog, and fatigue [4]. Patients report a great impact on their quality of life and ability to work or even to manage their everyday life [5]. The first steps in distinguishing predictors for some long COVID symptoms were made [6]. However, it remains unclear which patients are at higher risk for a prolonged course of the disease and if there are diagnostic tools to objectify long COVID.

Even with effective and available vaccines in Germany [7], it will take effort for individuals and society to find their way back to a *normal* everyday life. An important aspect of everyday life, which was dramatically altered during phases of COVID-19 pandemic lockdown, was social participation. Studies have shown that social participation is associated with life satisfaction, self-esteem, and mental health, for example, in older persons [8] and adolescents [9]. Therefore, understanding how people directly and indirectly affected by COVID-19 resume social participation in the vaccine era is also relevant for understanding the pandemic’s effect upon community health. The assessment of social participation during the pandemic and the active involvement of those affected by long COVID in research endeavors may provide important information on how measures, communication, and care services can be optimized in the future to best suit the health needs of the community.

### Primary Aim

With the joint project, DEFEAT Corona (Defense Against COVID-19 Study), we aim to improve the understanding of the long COVID syndrome and the long-term effects of the pandemic in a large cohort. We want to analyze which syndromic forms exist in patients with long COVID and how care structures should be built to ensure patient-centered support. We will assess how patients with long COVID participate socially in comparison to others who are also experiencing the pandemic.

With this study, we aim to build an innovative, web-based, and patient-centered research infrastructure to support the new challenges of the vaccine era of the COVID-19 pandemic.

## Methods

### Aim, Design, and Setting of the Study

The main goal of the joint project, DEFEAT Corona, is to create an innovative, web-based, and patient-centered research structure. With this platform, we want to face the challenges of the vaccine area in Lower Saxony, Germany. The 3 partners (Hannover Medical School, University Medical Center Göttingen, and Ostfalia University of Applied Sciences) plan to build a digital platform for long-term research in times of a global pandemic. There are 3 subprojects, which have the following primary and secondary objectives. Key to the project is to assess the long-term consequences of a SARS-CoV-2 infection and the pandemic in a prospective cohort study. This will be addressed in the 3 subprojects (Textbox 1).

**Primary and secondary aims of DEFEAT Corona (Defense Against COVID-19 Study).**

**Subproject 1: Back to life? Social and participation convalescence in the vaccine era**

- Analysis of how social participation is experienced by patients who had COVID-19 and have long term symptoms (patients with long COVID) vs persons who were affected by pandemic regulations but not infected by the virus
- Foundation of a patient- and public-led advisory board

**Subproject 2: COVID-19 special consultation Lower Saxony—vaccination response and long COVID syndrome**

- Establishment of a web-based, multimodal research platform and development of a self-assessment for patients with long COVID
- Setup of an innovative and transregional special consultation service for patients with long COVID syndrome (clinical care and research)
  Finding objective measures for the diagnosis of long COVID (eg, optical coherence tomography angiography imaging and ear, nose, and throat examination)
  Assessment of the needs of patients with long COVID in health care

**Subproject 3: machine learning for finding symptom clusters (MACLEAF SYCL)**

- Data preparation for exploratory analysis and definition of criteria for cluster analysis
- Implementation of the cluster analysis to identify symptom clusters with possible adjustments or extensions

**Overriding secondary objectives**

- A transparent communication of new insights to a nonprofessional audience
- Sustainable transfer of knowledge
- Making research participation accessible for parts of the population living further away from university cities.

### Characteristics of the Participants

Participants will be recruited via newspaper announcements, home page, posters, and flyers in local regions, general practices, or long COVID support groups in the northern German region of Lower Saxony. Furthermore, participants will be recruited in the outpatient clinics of the Hannover Medical School and University Medical Center Göttingen, through the cooperation partners, local public health authorities, primary care clinics, and ministries.

Everyone older than 18 years who can give informed consent is invited to participate in the first baseline web-based questionnaire, whether they had COVID-19 (with or without long-term effects) or not. We will also assess whether the participants live in Lower Saxony. Exclusion criteria are being younger than 18 years and refusal or inability to provide consent. Participants for subproject 2 will be recruited from the participants of the baseline questionnaire. Subproject 3 will analyze data assessed in the baseline questionnaire.

### Ethics Approval and Consent to Participate

The study is registered in the German clinical trial registry (DRKS00026007) and has been approved by the institutional review board of both Hannover Medical School (9948_BO_K_2021) and University Medical Center Göttingen (29/3/21). Data security management plan has also been approved by Hannover Medical School. Informed consent will be obtained from all study participants subsequently in each study step (questionnaires, clinical assessment and specimen collection, and qualitative study). Each participant receives written information on study procedures and data management over the web-based questionnaire format.

Study participation is voluntary, and participants have the right to withdraw consent at any time and without disclosure of reasons for withdrawal. A trained member of the study team is available for questions at enrollment and thereafter.

In the further steps of the study, participants will be informed about specimen and data collection, as well as the storage of samples in the Hannover Biobank for future research projects. Study participation is voluntary, and participants have the right to withdraw consent at any time and without disclosure of reasons for withdrawal. Furthermore, they will receive a written data security protocol. A trained member of the study team is available for questions at enrollment and thereafter. All collected data will be pseudonymized and stored on servers of Hanover Medical School or University Medical Center Göttingen. Publications will include anonymized data. Blood samples will be transferred to the Biobank of Hanover Medical School.

### Description of All Processes, Interventions, Comparisons

**Figure 1:**
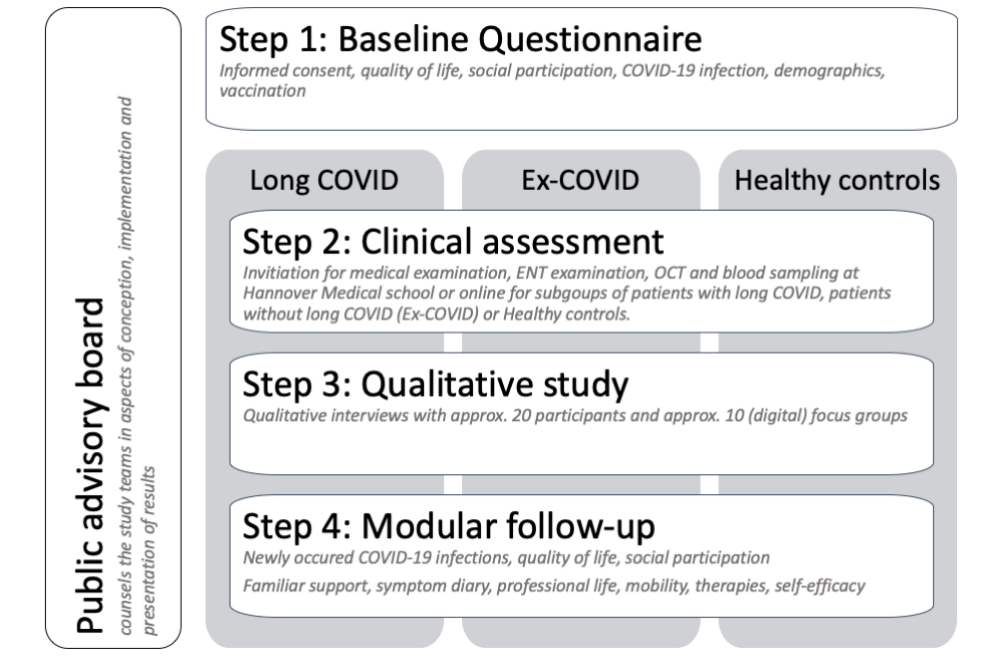
Flow chart of DEFEAT Corona (Defense Against COVID-19 Study). ENT: ears, nose, and throat; OCT: optical coherence tomography.

The details of the study design are illustrated in Figure 1.

#### Step 1: Baseline Questionnaire

The baseline questionnaire will include sociodemographic questions, questions about a SARS-CoV-2 infection, the process of the disease, possible late symptoms, and vaccination status. According to the participants’ answers in the basic questionnaire, they will be assigned into 3 groups: “No COVID,” “Ex-COVID,” and “Long COVID.” Questionnaire items were adapted from the World Health Organization Case Report Form [10] and the UK Long COVID guideline [11]. We also assessed quality of life (EQ-5D [12]) and social participation (IMET [13]; Multimedia Appendix 1).

The data of the baseline questionnaire will also be used in the cluster analysis to characterize the typical symptom complexes experienced by participants with long COVID.

#### Step 2: Obtaining Additional Clinical Characteristics in Ex-COVID and Long COVID Groups

A portion of the “Long COVID,” “Ex-COVID,” and “No COVID” groups will be invited for a personal clinical assessment to the outpatient clinic of Hanover Medical School. The clinical assessment will include blood sampling of 20-30 ml (eg, full blood count, electrolytes, liver enzymes, retention parameters, and SARS-CoV-2 antibodies), an ears, nose, and throat examination, optical coherence tomography angiography imaging, and a standardized medical examination by a trained physician. Some patients with long COVID will be invited to an additional web-based consultation.

#### Step 3: Qualitative Study of the Long COVID Illness Experience

According to the sociodemographic data gathered in the baseline questionnaire, a diverse group of participants with long COVID will be invited to participate in the qualitative arm of the study. We aim to perform 20-30 individual interviews and 10-20 focus groups to gain a deeper understanding about how people with various long COVID symptoms experience their illness, and how they perceive the illnesses impact on social participation. This includes relationships and activities among friends and family, education, profession, everyday life, health system, and leisure activities.

#### Step 4: Individualized, Modular, Follow-up Questionnaires

All participants will get invitations for modular questionnaires, depending on their answer in the baseline questionnaire (eg, how their children experienced the pandemic, if the participants stated that they had children). The follow-up questionnaires will contain questions about new infections with SARS-CoV-2, changes in symptoms and recovery from symptoms, further vaccinations, quality of life (EQ-5D), and social participation (IMET) over a longer period. The project will be accompanied by a patient- and public-led advisory board.

### Type of Data and Analyses Planned

Data preparation will be performed in SPSS (IBM Corp), data analysis in R (version 4.1.1; The R Foundation), and visualizations with GraphPad Prism (GraphPad Software Inc) and R. Types of data are mainly ordinal or numerical and sometimes strings of a free text, depending on the questions. Optical coherence tomography images will be analyzed with MATLAB (MathWorks), and values will be created for vessel density and vessel distance. During the data preparation, data quality will be checked, and the handling of missing values will be addressed. Summary statistics and standard visualizations will be computed. Within the collected data, symptom clusters after an infection with SARS-CoV-2 will be found using machine learning methods. Established methods of cluster and symptom cluster analysis will be adapted and extended for the specific questionnaire. Logistic regression will be applied to identify specific risk factors for development of long COVID.

Semistructured interviews and focus groups will last about 45-75 minutes. Interview and focus group data will be recorded and transcribed according to the simplified rules of Kuckartz [14] and Dresing and Pehl [15] and subsequently analyzed using qualitative content analysis according to Mayring [16] and Kuckartz [14].

Our mixed-methods approach to exploring social participation includes data from standardized questionnaires and qualitative interviews and focus groups. These data will be combined or triangulated to gain a deeper understanding of the social participation and the illness experience of persons experiencing long COVID symptoms.

## Results

The study began in July 2021 with the preparation of the first questionnaire. The first participants were recruited in September 2021 via web-based questionnaire. Until July 2022, approximately 4500 participants were recruited via a web-based questionnaire. Recruiting will continue at least until the end of 2022. The first clinical consultations of patients with long COVID took place in January 2022 and will continue throughout the duration of the study.

The qualitative study began in September 2021 with a series of preliminary exploratory discussions with members of the target population to assess important subjects and develop an interview guideline. This guideline will be used in the main qualitative study. Interviews will be conducted and analyzed, and new participants will be enrolled according to the principle of theoretical sampling. The interviews started in January 2022 and will continue until targeted sample size is achieved.

## Discussion

### Principal Findings

With DEFEAT Corona, we aimed to develop an innovative web-based platform for research on the sequelae of the pandemic and SARS-CoV-2 infections. The 3 subprojects focus on social participation, characteristics of long COVID, and the exploration of data-driven methods. As of July 2022, the data acquisition is projected to continue at least until December 2022.

The web-based platform is available on the DEFEAT Corona website [17]. As of July 2022, approximately 4500 people participated in our first questionnaire, so we assume that the platform has good usability. The recruitment is ongoing. We developed a questionnaire to assess social participation during a pandemic [18], which will be tested within DEFEAT Corona.

### Strengths and Limitations

A limitation to the study is the method of recruiting. The first questionnaire is available on the web for the public. People who do not use web-based resources frequently (eg, older people) might not participate, which may introduce a selection bias. Nevertheless, we aimed to create a platform that is easily accessible for participants, as it is not required to travel to study centers. In addition, people who are affected by long COVID symptoms such as fatigue might be more likely to participate in the study when they can participate from the comfort of their own home instead of needing to visit a large university hospital to be enrolled in the study. Therefore, the participants of DEFEAT Corona will be a convenience sample, and we must take these factors into account, especially when relating the study findings to the population.

### Future Directions

With our study design, we aim to find characteristics and determinants of the long COVID syndrome. The web-based approach can be easily adapted to another place or another disease. With the mixed-methods approach, we aim to include different aspects that might be important in our analysis. This will help us distinguish important topics regarding the long-term effects of the SARS-CoV-2 infection and the global pandemic and guide the further development of our research agenda.

## Appendix

Multimedia Appendix 1 First questionnaire of the DEFEAT Corona study (German).

## Abbreviations

DEFEAT Corona: Defense Against COVID-19 Study

## Data Availability

Data sampling is still in process. The data sets generated during this study are available from the corresponding author on reasonable request.

## References

[1] Coronavirus Research Center - COVID-19 Dashboard. Johns Hopkins University & Medecine.

[2] Soriano JB, Murthy S, Marshall JC, Relan P, Diaz JV, WHO Clinical Case Definition Working Group on Post-COVID-19 Condition (2022). A clinical case definition of post-COVID-19 condition by a Delphi consensus. Lancet Infect Dis.

[3] Lopez-Leon S, Wegman-Ostrosky T, Perelman C, Sepulveda R, Rebolledo PA, Cuapio A, Villapol S (2021). More than 50 long-term effects of COVID-19: a systematic review and meta-analysis. Sci Rep.

[4] Koczulla AR, Ankermann T, Behrends U, Berlit P, Böing S, Brinkmann F, Franke C, Glöckl R, Gogoll C, Hummel T, Kronsbein J, Maibaum T, Peters EMJ, Pfeifer M, Platz T, Pletz M, Pongratz G, Powitz F, Rabe KF, Scheibenbogen C, Stallmach A, Stegbauer M, Wagner HO, Waller C, Wirtz H, Zeiher A, Zwick RH (2021). [S1 Guideline Post-COVID/Long-COVID]. Pneumologie.

[5] Seeßle J, Waterboer T, Hippchen T, Simon J, Kirchner M, Lim A, Müller B, Merle U (2022). Persistent Symptoms in Adult Patients 1 Year After Coronavirus Disease 2019 (COVID-19): A Prospective Cohort Study. Clin Infect Dis.

[6] Sudre CH, Murray B, Varsavsky T, Graham MS, Penfold RS, Bowyer RC, Pujol JC, Klaser K, Antonelli M, Canas LS, Molteni E, Modat M, Jorge Cardoso M, May A, Ganesh S, Davies R, Nguyen LH, Drew DA, Astley CM, Joshi AD, Merino J, Tsereteli N, Fall T, Gomez MF, Duncan EL, Menni C, Williams FMK, Franks PW, Chan AT, Wolf J, Ourselin S, Spector T, Steves CJ (2021). Attributes and predictors of long COVID. Nat Med.

[7] Barros-Martins J, Hammerschmidt SI, Cossmann A, Odak I, Stankov MV, Morillas Ramos G, Dopfer-Jablonka A, Heidemann A, Ritter C, Friedrichsen M, Schultze-Florey C, Ravens I, Willenzon S, Bubke A, Ristenpart J, Janssen A, Ssebyatika G, Bernhardt G, Münch Jan, Hoffmann M, Pöhlmann S, Krey T, Bošnjak B, Förster R, Behrens GMN (2021). Immune responses against SARS-CoV-2 variants after heterologous and homologous ChAdOx1 nCoV-19/BNT162b2 vaccination. Nat Med.

[8] Takagi D, Kondo K, Kawachi I (2013). Social participation and mental health: moderating effects of gender, social role and rurality. BMC Public Health.

[9] Santini ZI, Pisinger VSC, Nielsen L, Madsen KR, Nelausen MK, Koyanagi A, Koushede V, Roffey S, Thygesen LC, Meilstrup C (2021). Social Disconnectedness, loneliness, and mental health among adolescents in Danish high schools: a nationwide cross-sectional study. Front Behav Neurosci.

[10] Global COVID-19 Clinical Platform Case Report Form (CRF) for Post COVID condition (Post COVID-19 CRF). World Health Organization.

[11] Shah W, Hillman T, Playford ED, Hishmeh L (2021). Managing the long term effects of covid-19: summary of NICE, SIGN, and RCGP rapid guideline. BMJ.

[12] Rabin R, de Charro F (2001). EQ-5D: a measure of health status from the EuroQol Group. Ann Med.

[13] Deck R, Mittag O, Hüppe A, Muche-Borowski C, Raspe H (2011). IMET. Index zur Messung von Einschränkungen der Teilhabe. Leibniz-Zentrum für Psychologische Information und Dokumentation (ZPID).

[14] Kuckartz U, Rädiker S (2016). Qualitative Inhaltsanalyse. Methoden, Praxis, Computerunterstützung. 3. überarbeitete Auflage [Qualitative content analysis. Methods, practice, computer support. 3rd Edition].

[15] Dresing T, Pehl T (2015). Praxisbuch Interview, Transkription & Analyse:Anleitungen und Regelsysteme für qualitativ Forschende: Anleitungen und Regelsysteme für qualitativ Forschende. 6. Auflage [Interview, Transcription & Analysis Practice Book: Guidance and control systems for qualitative researchers. 6th Edition].

[16] Mayring P (2015). Qualitative Inhaltsanalyse, Grundlagen und Techniken. 12., aktualisierte und überarb. Aufl [Qualitative content analysis, basics and techniques. 12th Edition].

[17] DEFEAT Corona.

[18] Schröder D, Heesen G, Heinemann S, Hummers E, Jablonka A, Steffens S, Mikuteit M, Niewolik J, Overbeck TR, Kallusky J, Müller F (2022). Development and Validation of a Questionnaire to Assess Social Participation of High Risk-Adults in Germany During the COVID-19 Pandemic. Front Public Health.

