# Supplementary material for: Long-term Consequences of COVID-19 and the Pandemic: Protocol for a Web-Based, Longitudinal Observational Study (DEFEAT)
Source: JMIR Res Protoc. 2022 Oct 26;11(10):e38718. doi: 10.2196/38718 (PMC9611102; doi:10.2196/38718)
Supplement: Multimedia Appendix 1 [file resprot_v11i10e38718_app1.pdf]

EI01

Liebe Studienteilnehmer:innen,

mit dem neuen Projekt DEFEAT Corona soll das Long COVID Syndrom weiter erforscht werden. Das Projekt wird von der Medizinischen Hochschule Hannover, der Universität Göttingen und der Ostfalia Hochschule für angewandte Wissenschaften Wolfenbüttel durchgeführt und wird von der EU (EFRE) gefördert.

Wir laden alle Menschen über 18 Jahre zur Teilnahme an der Befragung ein. Wir suchen Sie, wenn Sie:

- keine Corona Infektion (als Vergleichsgruppe),
- eine Corona Infektion oder
- Spätsymptome nach einer Corona Infektion hatten oder haben.

Vielen Dank für Ihre Teilnahme! Wir bitten Sie, den Link für die Umfrage an Freund:innen, Familie und Bekannte weiterzuleiten. Jede Teilnahme hilft uns, das Long COVID Syndrom besser zu verstehen und können die Behandlung und das weitere Outcome von Corona Patient:innen positiv beeinflußen.

https://webext.mh-hannover.de/soscisurvey/defeatcorona1/?q=dftc

Noch Fragen?
Wir stehen Ihnen unter info@defeat-corona.de gerne zur Verfügung.

EI02

Was ist DEFEAT Corona?

In dem Projekt DEFEAT Corona wird untersucht, wie es Menschen geht, die eine COVID Infektion durchgemacht haben. Ca. 10% der Patient:innen, die Corona hatten, leiden unter Langzeitfolgen. Derzeit gibt es noch wenige Informationen zum sogenannten Long COVID Syndrom.

Wir laden alle Menschen, die älter als 18 Jahre sind, zur Teilnahme ein, egal ob Sie eine Corona Erkrankung hatten oder nicht. Im Fragebogen sind Fragen zu gesundheitlichen Beschwerden während, nach oder auch ohne eine Corona Infektion und zu Ihrer sozialen Situation, Vorerkrankungen und Lebensqualität.

Wir würden uns sehr freuen, wenn Sie uns ihre Kontaktdaten hinterlassen würden, damit wir Sie ggf. zu einer weiterführenden Untersuchung (Befragung, Interview, Spezialsprechstunde) einladen können. Dafür erhalten Sie dann gesonderte Informationen. (Teilnehmer:innen Information)

Die Teilnahme an dieser Studie ist freiwillig. Ihre Daten werden in pseudonymisierter Form auf dem Server des Rechenzentrums der MHH gemäß den datenschutzrechtlichen Vorschriften, insbesondere der DSGVO, gespeichert und verarbeitet. Weitere Informationen entnehmen Sie bitte dem Datenschutzkonzept (Datenschutzkonzept).

Eine Beendigung der Studienteilnahme ist jederzeit ohne Angabe von Gründen möglich. Im Falle eines Widerrufs werden Ihre Daten gelöscht, sofern Sie nicht erlauben, dass Ihre Daten in anonymisierter Form für die Studie weiterverwendet werden dürfen.

Haben Sie Fragen zur Studie?

Melden Sie sich gerne unter info@defeat-corona.de oder unter 0160 9779 1528.

EI05

Mit dem Absenden dieses Formulars bestätige ich:

- Ich habe die Teilnehmer:inneninformationen gelesen und verstanden. Wenn ich Fragen habe, wurden sie entweder per E-Mail oder telefonisch beantwortet.
- Ich nehme freiwillig teil. Ich kann jederzeit und ohne Angaben von Gründen meine Einwilligung widerrufen, ohne dass mir daraus irgendwelche Nachteile entstehen.
- Meine Daten werden nur für wissenschaftliche Zwecke von der MHH der UMG und der Ostfalia Hochschule ausgewertet.
- Meine Kontaktdaten werden gesondert gespeichert und nicht mit meinen Angaben in den Fragebögen vermischt (pseudonymisierte Datenverwaltung). Meine Kontaktdaten würden nicht weitergegeben.

○ Ja, ich möchte teilnehmen

○ Nein, ich möchte nicht teilnehmen

1 aktive(r) Filter

Filter EI05/F1
Wenn eine der folgenden Antwortoption(en) ausgewählt wurde: **2**
Dann nach dem Klick auf "Weiter" den Text **EI13** anzeigen und das Interview beenden

EI28

Erstellung eines Pseudonyms

Wir möchten Sie bitten ein Pseudonym für diese Studie zu erstellen. Das Pseudonym besteht aus Buchstaben und Zahlen.

Die Daten in der Studie werden nur mit dem Pseudonym verarbeitet, Sie sind damit nicht identifizierbar. Falls Sie weitere Fragebögen beantworten, können wir die Antworten miteinander verbinden, ohne persönliche Daten von Ihnen zu erheben. Bitte merken Sie sich Ihr Pseudonym, um es ggf. später wieder anzugeben.

Mehr über die Erstellung des Pseudonyms erfahren Sie hier.

EI08

Geburtsmonat der Mutter [Bitte auswählen]

EI09

Erster Buchstabe Vorname der Mutter [Bitte auswählen]

EI10

Geburtsmonat Vater [Bitte auswählen]

EI11

1. Buchstabe Vorname Vater [Bitte auswählen]

EI12

Letzte Ziffer des eigenen Geburtsjahres [Bitte auswählen]

PHP-Code

```
// PS1 prüfen und ggf. umcodieren;
if (value('EI08')=='-9') {$Kode1 = "--";
} else {
for ($i = 1; $i <= 9; $i++) {
    if (value('EI08')==$i) {$Kode1 = "0".$i;
}}}
if (value('EI08')=='10') {$Kode1 = "10";}
if (value('EI08')=='11') {$Kode1 = "11";}
if (value('EI08')=='12') {$Kode1 = "12";}

// PS2 prüfen und ggf. umcodieren;
if (value('EI09')=='-9') {$Kode2 = "-";
} else {
if (value('EI09')==1) {$Kode2 = "A";}
if (value('EI09')==2) {$Kode2 = "B";}
if (value('EI09')==3) {$Kode2 = "C";}
if (value('EI09')==4) {$Kode2 = "D";}
if (value('EI09')==5) {$Kode2 = "E";}
if (value('EI09')==6) {$Kode2 = "F";}
```

```
if (value('EI09')==7) {$Kode2 = "G";}
if (value('EI09')==8) {$Kode2 = "H";}
if (value('EI09')==9) {$Kode2 = "I";}
if (value('EI09')==10) {$Kode2 = "J";}
if (value('EI09')==11) {$Kode2 = "K";}
if (value('EI09')==12) {$Kode2 = "L";}
if (value('EI09')==13) {$Kode2 = "M";}
if (value('EI09')==14) {$Kode2 = "N";}
if (value('EI09')==15) {$Kode2 = "O";}
if (value('EI09')==16) {$Kode2 = "P";}
if (value('EI09')==17) {$Kode2 = "Q";}
if (value('EI09')==18) {$Kode2 = "R";}
if (value('EI09')==19) {$Kode2 = "S";}
if (value('EI09')==20) {$Kode2 = "T";}
if (value('EI09')==21) {$Kode2 = "U";}
if (value('EI09')==22) {$Kode2 = "V";}
if (value('EI09')==23) {$Kode2 = "W";}
if (value('EI09')==24) {$Kode2 = "X";}
if (value('EI09')==25) {$Kode2 = "Y";}
if (value('EI09')==26) {$Kode2 = "Z";}
}

// PS3 prüfen und ggf. umcodieren;
if (value('EI10')=='-9') {$Kode3 = "--";
} else {
for ($i = 1; $i <= 9; $i++) {
    if (value('EI10')==$i) {$Kode3 = "0".$i;
}}}
if (value('EI10')=='10') {$Kode3 = "10";}
if (value('EI10')=='11') {$Kode3 = "11";}
if (value('EI10')=='12') {$Kode3 = "12";}

// PS4 prüfen und ggf. umcodieren;
if (value('EI11')=='-9') {$Kode4 = "-";
} else {
if (value('EI11')==1) {$Kode4 = "A";}
if (value('EI11')==2) {$Kode4 = "B";}
if (value('EI11')==3) {$Kode4 = "C";}
if (value('EI11')==4) {$Kode4 = "D";}
if (value('EI11')==5) {$Kode4 = "E";}
if (value('EI11')==6) {$Kode4 = "F";}
if (value('EI11')==7) {$Kode4 = "G";}
if (value('EI11')==8) {$Kode4 = "H";}
if (value('EI11')==9) {$Kode4 = "I";}
if (value('EI11')==10) {$Kode4 = "J";}
if (value('EI11')==11) {$Kode4 = "K";}
if (value('EI11')==12) {$Kode4 = "L";}
if (value('EI11')==13) {$Kode4 = "M";}
if (value('EI11')==14) {$Kode4 = "N";}
if (value('EI11')==15) {$Kode4 = "O";}
if (value('EI11')==16) {$Kode4 = "P";}
if (value('EI11')==17) {$Kode4 = "Q";}
if (value('EI11')==18) {$Kode4 = "R";}
if (value('EI11')==19) {$Kode4 = "S";}
if (value('EI11')==20) {$Kode4 = "T";}
if (value('EI11')==21) {$Kode4 = "U";}
if (value('EI11')==22) {$Kode4 = "V";}
if (value('EI11')==23) {$Kode4 = "W";}
if (value('EI11')==24) {$Kode4 = "X";}
if (value('EI11')==25) {$Kode4 = "Y";}
if (value('EI11')==26) {$Kode4 = "Z";}
}

// PS5 prüfen und ggf. umcodieren;
if (value('EI12')=='-9') {$Kode5 = "-";
} else {
for ($i = 1; $i <= 9; $i++) {
    if (value('EI12')==$i) {$Kode5 = $i;
}}}
```

```
if (value('EI12')=='10') {$Kode5 = "0";}

// Pat-ID aus Einzelvariablen zusammenstellen;
put('patcode',$Kode1.$Kode2.$Kode3.$Kode4.$Kode5);
```

Seite 05

EI29

Kontaktdaten angeben

Mit dieser Studie möchten wir über einen längeren Zeitraum Daten erheben, um bessere Erkenntnisse über Long COVID zu erlangen. Dafür möchten wir Sie ggf. erneut kontaktieren, um Ihnen weitere Fragebögen oder eine Einladung zu unserer Sprechstunde zu schicken.

Wir würden uns sehr freuen, wenn Sie Ihren Namen, eine Telefonnummer oder Mailadresse und das Pseudonym (von der letzten Seite) abgeben würden. Das Pseudonym benötigen wir, um die Fragebögen für Sie zu personalisieren.

Die Kontaktdaten werden getrennt von den anderen Fragebogenantworten gespeichert.

EI04

Hierfür gebe ich folgende Kontaktdaten an:

- ☐ Name, Email-Adresse, Telefonnummer und Pseudonym

Seite 06

SY03

Hatten Sie bereits eine Corona Erkrankung?

- ☐ Nein.
- ☐ Ja, durch einen PCR Test bestätigt (Abstrich, PCR Labortest): Datum des positiven Tests ________
- ☐ Ja, durch einen Antikörper Test bestätigt (Blutabnahme).
- ☐ Ja, durch einen Antigen-Schnelltest bestätigt (Abstrich, Ergebnis innerhalb von Minuten).
- ☐ Ich denke ja, aber es wurde nicht getestet.
- ☐ Sonstiges: ________

1 aktive(r) Filter

Filter SY03/F1
Wenn eine der folgenden Antwortoption(en) ausgewählt wurde: **2, 3, 4**
Dann Seite(n) **sym, symlc** des Fragebogens anzeigen (sonst ausblenden)

SY04

Wann traten die ersten Corona-Symptome auf bzw. wann haben Sie gemerkt, dass Sie Corona haben?
Bitte tragen Sie das ungefähre Datum ein.

[ ]

SY05

Wie stark waren Ihre Symptome während der Corona Infektion?

Symptome während der Corona Infektion

Keine Symptome (0)	1	2	3	4	5	6	7	8	9	Stärkste Symptome (10)
○	○	○	○	○	○	○	○	○	○	○

SY06

Wurden Sie im Krankenhaus behandelt?

- ☐ Nein
- ☐ Ja, auf einer Normalstation
- ☐ Ja, auf einer Intensivstation

SY07

Wie war der Verlauf der Erkrankung?

- ○ Ich bin wieder vollständig genesen, ich fühle mich wieder gesund.
- ○ Ich hatte länger als 4 Wochen Symptome und fühle mich jetzt wieder gesund.
- ○ Ich fühle mich nicht wieder gesund, ich habe weiterhin Symptome.

1 aktive(r) Filter

Filter SY07/F1
Wenn eine der folgenden Antwortoption(en) ausgewählt wurde: **1**
Dann nach dem Klick auf "Weiter" direkt zur Seite **impf1** springen

SY09

Welche Symptome erleben Sie seit der COVID Erkrankung (erstmalig nach Erkrankung aufgetreten)?
(Mehrfachauswahl möglich)

	Keine Symptome (0)										Stärkste Symptome (10)	Weiß ich nicht
Atemnot	○	○	○	○	○	○	○	○	○	○	○	○
	Keine Symptome (0)										Stärkste Symptome (10)	Weiß ich nicht
	○	○	○	○	○	○	○	○	○	○	○	○

Husten

Keine Symptome (0) | Stärkste Symptome (10) | Weiß ich nicht

Herzrasen

Keine Symptome (0) | Stärkste Symptome (10) | Weiß ich nicht

Engegefühl in der Brust

Keine Symptome (0) | Stärkste Symptome (10) | Weiß ich nicht

Schmerzen in der Brust

Keine Symptome (0) | Stärkste Symptome (10) | Weiß ich nicht

Fatigue/Erschöpfung

Keine Symptome (0) | Stärkste Symptome (10) | Weiß ich nicht

Fieber

Keine Symptome (0) | Stärkste Symptome (10) | Weiß ich nicht

Schmerzen

Keine Symptome (0) | Stärkste Symptome (10) | Weiß ich nicht

Konzentrationsschwäche, „Brain fog“, Vergesslichkeit

Keine Symptome (0) | Stärkste Symptome (10) | Weiß ich nicht

Kopfschmerzen

Keine Symptome (0) | Stärkste Symptome (10) | Weiß ich nicht

Schlafstörungen

Keine Symptome (0) | Stärkste Symptome (10) | Weiß ich nicht

Gefühlsstörungen an Armen und Beinen (Kribbeln, Nadelstiche, ...)

Keine Symptome (0) | Stärkste Symptome (10) | Weiß ich nicht

Schwindel

Keine Symptome (0) | Stärkste Symptome (10) | Weiß ich nicht

Verwirrtheit

Keine Symptome (0) | Stärkste Symptome (10) | Weiß ich nicht

Bauchschmerzen

Keine Symptome (0) | Stärkste Symptome (10) | Weiß ich nicht

	Keine Symptome (0)	Stärkste Symptome (10)	Weiß ich nicht
Übelkeit			
Durchfall			
Appetitlosigkeit			
Gelenkschmerzen			
Muskelschmerzen			
Depressive Symptome			
Angst Symptome			
Tinnitus			
Ohrenschmerzen			
Trockener/Rauer Hals			
Verlust des Geschmacks-/ Geruchsinns			
Hautausschläge			
Sonstige			

SY10

Wenn sonstige, welche?

SY08

Wann traten die Symptome nach der Corona Erkrankung auf?

Bitte tragen Sie das ungefähre Datum ein.

SY11

Hier haben Sie die Möglichkeit, Ihre Symptome und den Krankheitsverlauf genauer zu schildern, falls Sie Corona haben oder hatten.

Seite 09

impf1

IM01

Sind Sie gegen SARS-CoV-2 geimpft?

- Ja
- Nein
- Unbekannt

1 aktive(r) Filter

Filter IM01/F1
Wenn eine der folgenden Antwortoption(en) ausgewählt wurde: **1**
Dann Seite(n) **impf** des Fragebogens anzeigen (sonst ausblenden)

IM04

Erste Impfung

Datum der ersten Impfung

IM02

Impfstoff:

- Biontech/Moderna
- Astra Zeneca
- Johnson&Johnson
- Anderer

IM05

Zweite Impfung

Datum der zweiten Impfung

IM03

Impfstoff:

- Biontech/Moderna
- Astra Zeneca
- Johnson&Johnson
- Anderer

IM06

Dritte Impfung

Datum der dritten Impfung

IM07

Impfstoff:

- Biontech/Moderna
- Astra Zeneca
- Johnson&Johnson
- Anderer

DM14

Nun folgen einige Fragen zu ihrer Person.

DM01

Was ist ihr höchster Schulabschluss?

- ◯ keiner/Volksschulabschluss
- ◯ Hauptschulabschluss
- ◯ Realschulabschluss/POS (Mittlere Reife)
- ◯ Abitur/Fachhochschulreife

DM02

Leben, arbeiten, wohnen oder haben Sie ihre medizinische Versorgung in Niedersachsen?

- ◯ Ja
- ◯ Nein

DM03

Welchem Geschlecht fühlen Sie sich zugehörig?

- ◯ Weiblich
- ◯ Männlich
- ◯ Divers

DM04

Wie alt sind Sie?

[ ] Jahre

DM13

Leben Kinder unter 18 Jahren in Ihrem Haushalt?

- ◯ Ja
- ◯ Nein

DM05

Sind Sie zurzeit berufstätig?

- Ja, regelmäßig vollzeitbeschäftigt
- Ja, regelmäßig teilzeitbeschäftigt (≥ 15 Stunden/Woche)
- Ja, geringfügig oder unregelmäßig teilzeitbeschäftigt (< 15 Stunden/Woche)
- Nein (z.B. in Ausbildung, Rentner:in, Elternzeit...)

DM06

Sind Sie in einem Gesundheitsberuf tätig?

- Ja
- Nein

DM07

Sind Sie in der Patient:innenversorgung tätig?

- Ja
- Nein

DM08

Sind Sie in Deutschland geboren?

- Ja
- Nein, in einem anderen EU Land
- Nein, in einem Land außerhalb der EU

DM09

Was trifft auf Sie zu? (Mehrfachauswahl möglich)

- [ ] Ich bin deutsche:r Staatsbürger:in
- [ ] Ich bin nach 1949 nach Deutschland zugewandert
- [ ] Ein Elternteil oder beide Eltern ist/sind nach 1949 nach Deutschland zugewandert

DM10

Haben Sie Vorerkrankungen?

- Nein
- Ja

DM11

Falls Ja, welche? (Mehrfachauswahl möglich)

- ☐ Bluthochdruck
- ☐ Herzinsuffizienz
- ☐ Koronare Herzkrankheit
- ☐ Vorhofflimmern/-flattern
- ☐ andere Herzrhythmusstörungen
- ☐ Diabetes Typ 1
- ☐ Diabetes Typ 2
- ☐ Asthma Bronchiale
- ☐ COPD
- ☐ Niereninsuffizienz
- ☐ Gicht
- ☐ Gallensteinleiden
- ☐ starkes Übergewicht
- ☐ Morbus Crohn / Colitis Ulcerosa
- ☐ Chronische Hepatitis
- ☐ HIV
- ☐ Schuppenflechte / Psoriasis
- ☐ chronische Wunden
- ☐ Allergien
- ☐ Neurodermitis
- ☐ Schilddrüsenerkrankung
- ☐ Rheuma
- ☐ Polymyalgie / Polymyalgia rheumatica
- ☐ andere Autoimmunerkrankungen
- ☐ chronische Schmerzen
- ☐ Migräne
- ☐ Epilepsie/Anfallsleiden
- ☐ Parkinson
- ☐ Demenz
- ☐ Schizophrenie/Manie
- ☐ Depression
- ☐ Durchblutungsstörung der Beine (pAVK)
- ☐ sonstige Erkrankung: ____________________

DM12

Falls ja, haben oder hatten Sie eine der folgenden Krebserkrankungen? (Mehrfachauswahl möglich)

- ☐ Nein
- ☐ Lungenkrebs
- ☐ Hautkrebs
- ☐ Brustkrebs
- ☐ Darmkrebs
- ☐ Magenkrebs
- ☐ Prostatakrebs
- ☐ andere/sonstige ____________________

EQ01

EuroQoL

Bitte tippen Sie auf den folgenden Bildschirmseiten die Aussage an, die Ihre Gesundheit HEUTE am besten beschreibt.

EQ02

Beweglichkeit/Mobilität HEUTE

- ○ Ich habe keine Probleme herumzugehen
- ○ Ich habe einige Probleme herumzugehen
- ○ Ich bin ans Bett gebunden

EQ03

Für sich selbst sorgen HEUTE

- ○ Ich habe keine Probleme, für mich selbst zu sorgen
- ○ Ich habe einige Probleme, mich selbst zu waschen oder mich anzuziehen
- ○ Ich bin nicht in der Lage, mich selbst zu waschen oder anzuziehen

EQ04

Alltägliche Tätigkeiten HEUTE
(z. B. Arbeit, Studium, Hausarbeit, Familien- oder Freizeitaktivitäten)

- ○ Ich habe keine Probleme, meinen alltäglichen Tätigkeiten nachzugehen
- ○ Ich habe einige Probleme, meinen alltäglichen Tätigkeiten nachzugehen
- ○ Ich bin nicht in der Lage, meinen alltäglichen Tätigkeiten nachzugehen

EQ05

Schmerzen/Körperliche Beschwerden HEUTE

- ○ Ich habe keine Schmerzen oder Beschwerden
- ○ Ich habe einige Schmerzen oder Beschwerden
- ○ Ich habe extreme Schmerzen oder Beschwerden

EQ06

Angst/Niedergeschlagenheit HEUTE

- ◯ Ich bin nicht ängstlich oder deprimiert
- ◯ Ich bin mäßig ängstlich oder deprimiert
- ◯ Ich bin extrem ängstlich oder deprimiert

EQ08

Gesundheitszustand

• Wir wollen herausfinden, wie gut oder schlecht Ihre Gesundheit HEUTE ist.

• Hier sehen Sie eine Skala, die mit Zahlen von 0 bis 100 versehen ist.

• 100 ist die beste Gesundheit, die Sie sich vorstellen können.
0 (Null) ist die schlechteste Gesundheit, die Sie sich vorstellen können.

• Bitte tippen Sie den Punkt auf der Skala an, der Ihre Gesundheit HEUTE am besten beschreibt.

	schlechtester Gesundheitszustand (0)	beste Gesundheits (100
Ihr heutiger Gesundheitszustand		

© EuroQol Group. EQ-5D™ is a trade mark of the EuroQol Group

EQ09

ME01

Die folgenden Fragen beziehen sich auf mögliche Beeinträchtigungen im Alltag durch Erkrankungen und die Pandemiesituation.

Bitte kreuzen Sie bei jeder Frage das entsprechende Kästchen an. Dabei bedeuten:

[0]= keine Beeinträchtigung ... ... [10] = keine Aktivität mehr möglich

Ansonsten kreuzen Sie ein entsprechendes Kästchen dazwischen an.

**Bestehen Beeinträchtigungen bei...**	keine Beeinträchtigung (0)										keine Aktivität mehr möglich (10)
üblichen Aktivitäten des täglichen Lebens (dieser Bereich bezieht sich auf Tätigkeiten wie z.B. Waschen, Ankleiden, Essen, sich im Haus bewegen, etc.)	○	○	○	○	○	○	○	○	○	○	○
familiären und häuslichen Verpflichtungen (dieser Bereich bezieht sich auf Tätigkeiten, die das Zuhause oder die Familie betreffen. Er umfasst Hausarbeit und andere Arbeiten rund um das Haus bzw. die Wohnung, auch Gartenarbeit)	○	○	○	○	○	○	○	○	○	○	○
Erledigungen außerhalb des Hauses (dieser Bereich umfasst z.B. Einkäufe, Amtsgänge, Bankgeschäfte auch unter Benutzung üblicher Verkehrsmittel)	○	○	○	○	○	○	○	○	○	○	○
täglichen Aufgaben und Verpflichtungen (dieser Bereich umfasst alltägliche Aufgaben und Verpflichtungen wie z.B. Arbeit, Schule, Hausarbeit)	○	○	○	○	○	○	○	○	○	○	○
Erholung und Freizeit (dieser Bereich umfasst Hobbys, Freizeitaktivitäten und Sport, Urlaub)	○	○	○	○	○	○	○	○	○	○	○
sozialen Aktivitäten (dieser Bereich bezieht sich auf das Zusammensein mit Freunden und Bekannten, wie z.B. Essen gehen, besondere Anlässe, Theater- oder Kinobesuche, etc.)	○	○	○	○	○	○	○	○	○	○	○
engen persönlichen Beziehungen (dieser Bereich bezieht sich auf Eingehen und Aufrechterhalten enger Freundschaften, Partnerschaften, Ehe)	○	○	○	○	○	○	○	○	○	○	○
Sexualleben (dieser Bereich bezieht sich auf die Häufigkeit und die Qualität des Sexuallebens)	○	○	○	○	○	○	○	○	○	○	○

ME02

**Wie stark sind folgende Belastungen?**	kann Belastungen ertragen										kann Belastungen nicht mehr ertragen
Stress und außergewöhnliche Belastungen (dieser Bereich umfasst z.B. familiäre Auseinandersetzungen und andere Konflikte sowie außergewöhnliche Belastungen im Beruf und am Arbeitsplatz)	○	○	○	○	○	○	○	○	○	○	○
Seelische Belastungen durch Pandemiesituation (z.B. Stimmungsschwankungen, Ärger, Depressionen, Angst o.ä.)	○	○	○	○	○	○	○	○	○	○	○

IMET 2006 © Deck, R., Borowski, C., Mittag, O., Hüppe, A., Raspe, H.

ME03

Vielen Dank für Ihre Teilnahme!

Wir möchten uns ganz herzlich für Ihre Mithilfe bedanken.

Ihre Antworten wurden gespeichert, Sie können das Browser-Fenster nun schließen.
